# Expert risk perceptions and the social amplification of risk: A case study in invasive tree pests and diseases

**DOI:** 10.1016/j.envsci.2017.08.020

**Published:** 2017-11

**Authors:** Julie Urquhart, Clive Potter, Julie Barnett, John Fellenor, John Mumford, Christopher P. Quine

**Affiliations:** a Centre for Environmental Policy, Faculty of Natural Sciences, Imperial College London, South Kensington Campus, London, SW7 1NA, United Kingdom; b Department of Psychology, University of Bath, 10West, Bath, BA2 7AY, United Kingdom; c Forest Research, Northern Research Station, Roslin, Midlothian, EH25 9SY, United Kingdom

**Keywords:** Social amplification of risk, Expert risk perceptions, Tree health, Ash dieback, Oak Processionary moth

## Abstract

The Social Amplification of Risk Framework (SARF) is often used as a conceptual tool for studying diverse risk perceptions associated with environmental hazards. While widely applied, it has been criticised for implying that it is possible to define a benchmark ‘real’ risk that is determined by experts and around which public risk perceptions can subsequently become amplified. It has been argued that this objectification of risk is particularly problematic when there are high levels of scientific uncertainty and a lack of expert consensus about the nature of a risk and its impacts. In order to explore this further, this paper examines how ‘experts’ – defined in this case as scientists, policy makers, outbreak managers and key stakeholders – construct and assemble their understanding of the risks associated with two invasive tree pest and disease outbreaks in the UK, ash dieback and oak processionary moth. Through semi-structured interviews with experts in each of the case study outbreaks, the paper aims to better understand the nature of information sources drawn on to construct perceptions of tree health risks, especially when uncertainty is prevalent. A key conclusion is that risk assessment is a socially-mediated, relational and incremental process with experts drawing on a range of official, anecdotal and experiential sources of information, as well as reference to past events in order to assemble the risk case. Aligned with this, experts make attributions about public concern, especially when the evidence base is incomplete and there is a need to justify policy and management actions and safeguard reputation.

## Highlights

• Expert risk perceptions are socially-mediated, relational and incremental.
• Challenges experts’ ‘real’ risk around which public perceptions are amplified.
• Tree health risks characterised by uncertainty and conflicts about what is at risk.
• Experts attribute concern to publics as they assemble risk judgments.
• Risk managers deal with the ‘risk event’ alongside ‘the social construction of risk’.

## 1 Introduction

In recent decades there has been a dramatic increase in new tree pest and disease epidemics, a development closely linked to globalization, trade in plant material and wood packaging and human-induced climate change (Potter and Urquhart, 2017). The technical process of identifying the risks associated with new and emerging tree and plant pests – a Pest Risk Analysis (PRA) – is used to determine appropriate phytosanitary measures and assess the likely biological, economic and social impacts of the outbreak (FAO, 2013). However, these assessments often have to deal with large degrees of uncertainty, particularly when scientific evidence is lacking, inconclusive or emerges piecemeal as outbreaks unfold (DEFRA, 2014, Barnett and Weyman, 2015). Developing a PRA may involve extrapolating existing data from other geographical locations where the pest is present and where the climatic and ecological conditions may be quite different. Further, the interaction with broader issues such as global trade and climate change means that finding solutions acceptable to all stakeholders is often problematic and costly, presenting a challenge to decision-makers about how best to address the issue when there is significant divergence in the risk understandings of different groups (Busby et al., 2009).

Understanding the underlying social and cultural processes that help shape differing, and sometimes conflicting, perceptions of environmental hazards have been the focus of much research on risk perception. Much of this work concentrates on the reasons for, and implications of, differences or discrepancies in the way experts and lay publics perceive risk (Busby and Duckett, 2012). In such studies, the Social Amplification of Risk Framework (SARF) (Kasperson et al., 1988) is often used as a point of reference to help explain how the risk perceptions of lay publics can sometimes diverge from those of experts, either intensifying or attenuating the risk in a process of ‘social amplification’ (Lazo et al., 2000, Savadori et al., 2004, Sjöberg and Drottz-Sjöberg, 1993, Kasperson, 2012). The implication is that lay public perceptions of the risk are effectively being judged against a ‘real’ or benchmark expert risk assessment (Merkelsen, 2011). There is an assumption, grounded in early risk research, that expert perceptions of risk are objectively based on technical risk estimates, in contrast to lay perceptions which are more complex and reflect a number of qualitative characteristics such as ‘dread’ or ‘familiarity’ (Slovic et al., 1979, Fischhoff et al., 1978, Sjöberg, 2002, Renn, 2004).

However, as Rowe and Wright (2001) concluded, there is very little empirical evidence to support the assertion that experts judge risks any differently from lay publics or that the resulting expert risk assessments are more ‘objective’ in nature. Work by social scientists points to expert risk perceptions being just as likely as lay publics to be socially constructed and mediated through social filters such as personal worldviews, biases, institutional affiliations and personal experience (see, for instance, Sjöberg, 2002; Lynn, 1986). Likewise, cultural theorists argue that objective processes of risk analysis are a misnomer, with experts demonstrating bias in the information they draw on, despite attempts at objectivity (Duckett et al., 2015, Wynne, 1996, Shortall, 2013). Douglas (1992) concludes that quantitative risk analysis is, thus, inadequate, particularly for complex and contested environmental hazards and objectivity cannot be seen as an ‘escape route’ for risk policy. Further, the notion of an objective expert evaluation of the ‘real’ risk is particularly problematic and empirically difficult to validate where there are high levels of scientific uncertainty and where there are contested claims about the nature of the ‘real’ risk (Rayner, 1988, Busby et al., 2009, Busby and Onggo, 2012, Pidgeon and Barnett, 2013). Alongside this, the public and media may have legitimate concerns that go beyond technical risk assessments of probability and magnitude of harm (Merkelsen, 2011, Pidgeon and Barnett, 2013).

Thus, despite inclusion of feedback loops in SARF, the perceived linearity of its sender-message-receiver model has been criticised as leading to a simplified characterisation of a complex set of relationships that fails to fully recognise that experts as well as lay publics are social actors, observing each other’s responses, interacting and sometimes reassessing their own perceptions and understandings as a result (Rayner, 1988, Petts et al., 2001, Murdock et al., 2003, Merkelsen, 2011, Busby and Onggo, 2012). As Pidgeon and Barnett (2013) argue, it is important to understand *how* people interpret the information they receive just as much as looking at the routes through which risk information is transmitted.

That said, SARF’s concept of ‘social stations of amplification’ recognises that individuals do act as members of larger social units and may perceive risks through the values of the organisation or group to which they belong (or from which they receive their communications), together with any associated cultural biases (Kasperson, 2012; Dietz and Stern,1996). Yet while SARF assigns experts a pivotal role as ‘social stations of amplification’, the processes (other than assumed procedural and technical ones) through which the experts construct and justify their risk judgements are not well explored, particularly when there is insufficient scientific evidence and when there may be divergent assessments about the likely impacts and appropriate management response. The question of how risk signals are assembled, deliberated on and communicated by these actors over the course of a risk event thus deserves further investigation.

In this paper our aim is to explore how ‘experts’ construct their understanding of tree health risks, exemplified through two invasive tree pest and disease outbreaks in the UK, ash dieback (*Hymenoscyphus fraxineus*) and oak processionary moth (*Thaumetopoea processionea*). We consider the nature of uncertainty in each case and, following SARF, the information sources drawn on by experts – defined in this case as scientists, policy makers, outbreak managers and key stakeholders – to assess the risks and the cultural, psychological and institutional processes, heuristics and social relations that shape their risk perceptions. Following Busby and Duckett (2012), we analyse the way some experts working in this area appear to make assumptions about, and have assigned particular risk attributions to, the policymakers whom they are being called on to advise and the lay public whose behaviour is seen to be driving some of the risk issues. We argue that these attributions are an increasingly important part of the tree health risk narrative, with potentially significant consequences for the way in which a risk event is anticipated, managed and communicated. Before presenting the findings from our empirical case studies, the following section outlines the methods adopted.

## 2 Methods

The first task was to undertake a documentary analysis to review academic, policy and grey literature in order to outline the technical risk assessment process in each outbreak, along with the policy and management responses. An internet search was undertaken using key terms such as ‘ash dieback’, ‘chalara’, ‘fraxineus’, ‘oak processionary moth’ and ‘OPM’ to identify organisations and documents associated with each outbreak. Further searches were undertaken on websites of each identified organisation, collating material such as scientific reports, government reports (e.g. PRAs) and policy documents, minutes from meetings, industry and NGO publications, and House of Commons/Lords debates. The documentary review was further explored, corroborated and critiqued through semi-structured interviews (Creswell, 2013) with a range of experts for each outbreak between March and November 2015. Questions sought to elicit respondents’ own recollections of the outbreak and the focus of their risk concerns. We sought to understand whether respondents felt their perceptions of the risk had changed over time and the sources of information they drew on to make their judgements about the risk, as well as their beliefs about the risk concerns of other stakeholders and public.

Our sample was purposefully broad and we defined ‘experts’ as the scientists who made the initial judgements about risk in preparing a PRA, policymakers who have to decide how and when to act, staff of public organisations who deal directly with managing outbreaks and a wide array of key stakeholders (such as NGOs, the nursery sector and foresters) with whom they interact. Our rationale for this sample was to analyse how individuals across different scales and foci of the outbreak constructed their risk assessments. A long list of potential respondents who fitted the above criteria was identified as part of the documentary analysis. The final purposive sample of 37 individuals (ash dieback – 21; oak processionary moth (OPM) – 16) was selected through the professional knowledge of the project team and its advisory committee, along with snowball sampling where study respondents recommended potential additional respondents from their own professional networks (Montello and Sutton, 2013).

All interviews were conducted in person, lasting between 45 and 90 min, and were digitally recorded and transcribed verbatim. In line with research ethics requirements, all respondents participated voluntarily and have been anonymised throughout this paper, with respondent codes assigned. The transcripts were analysed using a combination of manual and digital coding (Nvivo 10.2 qualitative software) in a process of thematic analysis within a social constructionist epistemology (Creswell, 2013, Braun and Clarke, 2006). In the spirit of SARF, our analysis sought to identify the sources of information that experts draw on, alongside the affective and cognitive filters, heuristic devices and interactions with others (including the pest/disease as well as other social actors), which shape their perceptions.

In the following sections we present a narrative account of each outbreak, outlining the technical risk assessment process and how this is drawn on by decision-makers, alongside how arguments were made and justified over time by utilising information from a diverse set of sources to validate respondents’ perceptions of the risk.

## 3 Oak processionary moth

OPM is a native of southern Europe, but over recent decades its range has expanded northwards, with populations now established in north and west Europe (Groenen and Meurisse, 2012). The larvae of OPM can cause defoliation of oak trees, making them vulnerable to attack by other pests and diseases and to environmental factors such as drought. The caterpillars also present a threat to human and animal health. They have tiny hairs containing a toxin that can cause itching and irritation of the skin, eyes and respiratory system (Maier et al., 2003, Mindlin et al., 2012).

The pest was first discovered in the UK in 2006 on 20 newly planted fastigiate oaks (*Quercus robur* ‘Fastigiata’) in the car park of a new housing development in Richmond, West London. Shortly thereafter, a further outbreak was identified in Ealing, also in West London, on a stand of newly planted cypress oaks (*Quercus robur* ‘Fastigiata Koster’). The source of the infestation was traced to an import of amenity cypress oaks that had been grown in Italy and shipped to the UK from the Netherlands in 2004 (Potter et al., 2014).

From the outset there was a lack of consensus over which government agency should have overall responsibility for dealing with the outbreak (Tomlinson et al., 2015). While the Forestry Commission (FC) is responsible for the protection of forest trees and the Department for Environment, Food and Rural Affairs (Defra) has responsibility for nursery trees and imported stock (Tomlinson et al., 2015), protection of urban trees is a somewhat grey area (FC, 2011). The human health dimension, the remit of the Health Protection Agency (HPA), added further complexity to the already unclear regulatory framework. Discussions were held over the winter of 2006/2007 to decide which agency would take statutory responsibility, with the FC eventually taking the lead, and the HPA (now Public Health England) adopting a supportive role. The rationale for this decision was that it might prove difficult to argue for OPM control under public health legislation, and that more effective legal powers were likely under the Plant Health Act (1967). In any case, this delay in assigning regulatory responsibility inevitably impacted on the speed of response to the outbreak and thus the effectiveness of attempts to eradicate the pest (Tomlinson et al., 2015). Further, under the Plant Health (Forestry) Order 2005 the FC issues Plant Health Statutory Notices (PHSNs) to enforce management action on landowners or their agents, who are responsible for ensuring OPM nests are removed. However, this approach has been criticised as it depends on compliance from a large number of stakeholders and difficulties arise when there are high levels of non-compliance (Potter et al., 2014).

In line with standard practice when a new alien organism is detected, a PRA was conducted by scientific experts on behalf of the regulatory plant health authorities. According to the International Plant Protection Convention’s standards for phytosanitary measures, PRAs are designed to provide a systematic and objective assessment of the risk to identify pests of quarantine concern (IPPC, 2004). Thus, assembling the PRA involved identifying the likely pathways of introduction, outlining possible future impacts and recommending appropriate phytosanitary and management measures. Entomologists were aware of the impacts of OPM prior to its arrival in the UK through their academic and wider networks across Europe, outlining how OPM had contributed to oak decline in Germany (Möller, 2006) and human and animal health impacts in Belgium and the Netherlands (Gottschling and Meyer, 2006). These observations were drawn on in the PRA to conclude that OPM was likely to become established in the UK, although it was uncertain to what extent it might spread from the initial outbreak in West London. The PRA further concluded that there was a high degree of certainty that the most likely pathway for OPM entry into the UK was via ‘plants for planting’, especially the importation of semi-mature trees for ‘instant’ landscapes (Evans, 2007). The economic impact of OPM was considered high in terms of losses due to a decline in timber quality, but also potential tourism losses if the pest reduced the amenity value of oak-rich publicly accessible forests. In addition, the economic cost of controlling the pest because of human and animal health risks was also assessed as significant.

While the process of undertaking a PRA is intended to provide an objective assessment of the risks associated with a potential new pest, the scientists working on them also indicated to us the need to be pragmatic in their recommendations, keeping in mind the particularities of the EU plant health regime and international trade agreements under the World Trade Organisation Sanitary and Phytosanitary (SPS) Measures. These politico-economic factors contributed to the way experts and decision makers in our study framed the risk of OPM. However, as one scientist (R1) engaged in producing risk assessments asserted, even at the regulatory level in Europe, it was not clear-cut whether OPM should be framed as a tree or human health risk. Some member states (e.g. the Netherlands) considered OPM to be mainly a public health pest, while others saw it as predominantly a forest pest (e.g. Germany), depending on whether OPM was mainly impacting urban or forest trees. Explaining how any actions sanctioned under the British Plant Health Act (1967) need to be justified in the PRA, this respondent reflected: “We had to be very careful in the pest risk analysis that we kept the emphasis on the defoliation and issues of tree health, and only mention in passing, if you like, that it was also an issue with urticating hairs” (R1).

Thus, the OPM outbreak is characterised by high levels of certainty about its main entry pathway (plants for planting), but contestations over whether the pest should be considered primarily a tree health risk or a human/animal health risk. Responses in our study reflected this disagreement in risk perceptions. Perspectives ranged from considering OPM as a significant risk to a low risk, in relation to other threats. Five respondents were very concerned about OPM, feeling that it presents a significant risk to human, animal and tree health. Two managers of urban green spaces were further concerned about the potential impacts on the recreational use of open spaces, with one explaining: “If we’re going to end up with people saying, ‘actually I’m going to be resistant to going into these areas’, then that is appalling and tragic … woodland is a very important part of our natural environment and somewhere people need to be able to go and enjoy” (R2). A natural environment manager, however, disagreed, suggesting that “it won’t actually have a massive impact on how people use woods; it’s just another factor that people factor in when they go for a walk; they’re just aware of it, and it’s not going to stop people going out” (R3).

Two respondents, both health professionals, who felt that OPM has a low human health risk supported their position by suggesting that there is no evidence to suggest otherwise (e.g. no deaths, no anaphylaxis). This is reinforced by a Public Health England (PHE) systematic review of European literature, which concluded that OPM is a low risk to human health (O’Connell et al., 2015). In terms of PHE’s risk perceptions, a policy maker (P5) put this into context by indicating the need to balance the risks of OPM with other diseases: “We deal with meningitis and tuberculosis, where death and serious illness and significant public health… In our context it is a low public health risk. Rashes can be dealt with.”

Other respondents indicated that the current human and animal health risk from OPM is low because the pest is being actively managed. As a local authority manager (R4) commented: “If we’re not managing it, I can’t see how there wouldn’t be a massive outcry.” For others, such as environmental NGOs, attention coalesced around the potential impacts of the control methods (especially pesticides) used to treat OPM, rather than OPM itself. These responses echoed those outlined in a Butterfly Conservation report (BC, 2013), which expressed concerns over the effectiveness of prophylactic spraying, especially aerial spraying of woodland, and included concerns about the collateral damage on other species and the human health risks associated with the use of biopesticides.

Further, there was little consensus around whether OPM presents a significant risk to tree health. One outbreak manager (R5) said: “I think it’s a real challenge to suggest that oak trees will be severely, significantly affected by OPM. Individual trees, yes, I can see that. But population dynamics of any species is not going to want to destroy its habitat, so they go through peaks and troughs.”

Our analysis of the OPM case suggests that, rather than demonstrating an objective assessment of the risks, the institutional affiliation of respondents appeared to be a factor influencing how OPM and its attendant risks are framed. Unsurprisingly, those responsible for managing public spaces were most concerned about the risks to public health, while environmental organisations headlined the biodiversity impacts of the pesticides used to control the pest. For respondents who felt that OPM was a serious risk, anecdotal evidence was often drawn on to support their concerns. Reference was made, for instance, to cases of anaphylactic shock or the death of animals that they had heard about from Europe.

OPM is a pest that has the potential to affect people in terms of both direct health impacts and in limiting recreational use of the countryside and green spaces. Hence, respondents were surprised that OPM had not attracted more media attention, with only occasional news stories. Nevertheless, an outbreak manager expressed concern at some of the OPM media coverage though and worried that: “The news media put their own filter on the story which often comes out in the headlines like killer caterpillars … there are journalists out there who’ll use it as an anti-government, sort of, message or story. You know, the government’s fouled up again” (R6).

Following from this, reputational risk concerns were widely expressed. Many of the interviewed policymakers and risk managers were concerned about needing to be seen by wider publics and stakeholders as dealing with the outbreak appropriately and to “be seen to act reasonably” (R5). This suggests that, alongside dealing with the ‘real’ risk of the pest itself, risk managers also had to manage public (and other stakeholders) ‘perceived’ risk. As one of our respondents put it: “So there’s the actual, what is happening, in a true scientific view, but actually what is happening in people’s perceptions. And those are often quite far apart. And you somehow have to balance what’s the real risk, with actually what’s the perceived risk. And it’s managing that perceived risk, which I think is often difficult” (R5). This finding reflects Leiss’s (2003) observation that, in SARF terms, the ‘risk event’ (the objective characteristics of the hazard) and ‘the social construction of risk’ (concern about the hazard) present themselves to risk managers at the same time and must be attended to concurrently.

As our analysis has demonstrated, experts in the OPM case drew on a wide range of information sources besides official risk assessments to construct their understanding of the risk associated with the pest. This included personal experience of dealing with the outbreak, with one respondent reporting becoming sensitised to the caterpillars through direct exposure. Alongside official notifications about the pest, respondents used anecdotal evidence, particularly from outbreaks in other countries, to explain their perceptions of the risk. Institutional filters clearly had a role to play in shaping what respondents felt was ‘at risk’ and attributions were made about other stakeholders’ concerns. For risk managers, a need to protect the public from harm and to act responsibly in dealing with the outbreak influenced their perceptions of the risk and the resulting management strategies.

## 4 Ash dieback

Ash dieback is a fungal disease of ash trees caused by *Hymenoscyphus fraxineus*. It can infect many different species of ash, but the Common ash (*Fraxinus excelsior*) and Narrow-leaved ash (*Fraxinus angustifolia*) are the most severely affected, especially young trees (Kowalski, 2006, FR, 2012). The disease causes leaf loss, dieback of the crown and bark lesions. Infection with ash dieback is usually fatal, either directly or by weakening the tree, making it vulnerable to attack by other pests and pathogens. Natural spread is by wind-blown spores from the fruiting bodies (Queloz et al., 2011) that can result in the disease spreading up to 20–30 km per year (Solheim et al., 2011). In addition, the pathogen can be spread longer distances when infected plants are transported via trade pathways or when infected leaf litter is moved from an infected to an uninfected site. The first cases of ash dieback in Europe are believed to have originated in Poland, with infected trees reported in 1992 (Kowalski, 2006). Ash dieback is now widespread across Europe and in 2007 it was added to the European and Mediterranean Plant Protection Organization (EPPO) Alert List.

Ash dieback was first identified in the UK in February 2012 at a nursery in Buckinghamshire in a consignment of 600 trees from the Netherlands. Later that year it was confirmed in further nursery sites and in the wider environment. The government’s response was to issue a Plant Health Order (FC, 2012) on 29 October 2012, placing restrictions on importing ash trees into Britain and the emergency COBR(A) committee met to discuss how to deal with the spread of ash dieback. As a result, a rapid national survey of ash woodlands was undertaken over the following weekend and initial actions to tackle the threat were announced, which included tracing and destroying infected trees in nurseries and newly-planted sites (DEFRA, 2012). Scientific research was commissioned to improve understanding of the disease and identify tolerant variants, as well as the establishment of a Tree Health and Plant Biosecurity Expert Taskforce which aimed to assess the current disease threats to tree health more widely. While ash dieback is most severe in South East England and East Anglia, it has been identified across the UK and it is anticipated that it will kill a large number of ash trees in the coming decades. Current management involves efforts to slow the spread and scientific research to identify genetic variants that are tolerant to the disease (DEFRA, 2013).

Respondents in our study referenced the early scientific uncertainties concerning the identity and nature of the pathogen responsible for ash dieback. This, it has since been accepted, hindered early regulatory action to protect the UK from infection (Woodward and Boa, 2013). The fungus was first described in 2006 as *Chalara fraxinea* by Kowalski (2006). However, subsequent genetic and morphological research indicated that *Chalara fraxinea* is the asexual (anamorph) stage of the ascomycete fungus *Hymenoscyphus albidus*, known in Europe (including the UK) since 1851 and not considered pathogenic (Kowalski and Holdenrieder, 2009, Kraj et al., 2012). Thus, as one of the interviewed scientists explained: “We felt that as the organism was here and it was not doing any damage, as far as we could tell, to ash trees, and we thought it was widespread, then it couldn't be classified as a quarantine pest, and so we couldn’t put any statutory controls on it” (R7). By the time the biological identification of the pathogenic teleomorph was confirmed (Queloz et al., 2011), it was likely that the organism had already been present in the UK for several years prior to its discovery in 2012.

A PRA published in 2013 identified four main pathways of entry for ash dieback into the UK: plants for planting, wood, seeds and contaminated soil as a commodity or with host or non-host plants, with the importation of infected plants the likely main route of entry (Sansford, 2013). Estimates suggested that around half a million ash saplings were being imported every year (HCDEB, 2012). The PRA further acknowledged that natural spread through windblown spores from continental Europe was also being considered as a potential route of entry, citing modelling work undertaken by the University of Cambridge (DEFRA, 2013, Wentworth, 2012 Wentworth, 2012). An outbreak manager in our study supported this hypothesis: “[The fact that] the disease was present down the east coast meant that, without making a scientific statement of how it had got there, it was pretty obvious it was blowing in…. And people were saying it can’t cross the Channel… But it was all up the east coast. So how else did it get there then other than by natural means?” (R8). In contrast, a scientist respondent cited research by European plant pathologists (Chandelier et al., 2014) that concludes that long distance dispersal by air currents is unlikely: “The ascospores of ash dieback are very thin-walled and they're likely to desiccate fairly quickly after release…. the dispersal distance for the majority of ascospores of ash dieback is fairly short – we're talking about metres as opposed to kilometres” (R7).

Identifying the mode of entry of the pathogen into the UK was clearly a politically sensitive issue, not least in the context of growing media interest in ash dieback in 2012 which criticised the government for lax biosecurity and a failure to ban the import of ash early enough (Fellenor et al., under review; Heuch, 2014). For policy makers, pre-existing sensitivities to tree issues in the wake of the Government’s aborted attempt to sell off England’s public forest estate in 2010 may mean they were already sensitised to critical media coverage of a risk controversy associated with trees. As an outbreak manager (R8) explained to us: “In my view, the main driver was the media, and then the government response to the media.” Ash dieback was seen by politicians as a ‘national crisis’ (HOC, 2012), and an outbreak manager (R8) indicated that there was “strong pressure right from the very top for the government to be seen to be doing something about this.”

A representative of a forest industry body agreed that the Government response was partly influenced by a perceived public response, alongside lobbying from the environmental sector. In this regard, an industry representative described how the organisation, along with other NGOs, saw the ash dieback outbreak as an opportunity to raise tree health on the political agenda: “So we started getting some phone calls from the press about it and we very quickly decided that this was an opportunity for us to raise the whole profile of tree health within government circles. So we were very happy to brief the press and make it as big a story as possible, and as threatening” (R10).

The ash dieback case highlights some of the issues and contestations around the science-policy interface and how science and a risk case are used to inform policy or to justify policy decisions. Clearly, ash dieback had become a political issue with the government response reflecting what Hood (2007) calls a ‘negativity bias’, where risk managers pay more attention to negative rather than positive reactions in an effort to avoid blame and maintain reputation. Early uncertainty about the identity of the ash dieback pathogen meant that it was difficult to regulate for under European law, especially for an organism that had already been introduced into the Eurozone. One scientist spoke about the difficulty of giving advice to regulators when there was no scientific certainty about the identity of the fungus (R11). Several others, however, suggested that perhaps a precautionary approach should have been adopted meanwhile when it was recognised that a new pathogen was spreading across Europe.

Those dealing with the outbreak on the ground described how, early in the outbreak, at a stage when there was little unambiguous scientific evidence, they drew on a range of sources to make their risk judgements. For instance, a local authority representative (R12) indicated first hearing about ash dieback in the media, and that the level of news coverage made them think it was a serious issue. This led them to examine trees in the local area, where evidence of mature trees dying quickly did not seem to align with official assessments that older trees would probably survive for many years. The respondent was concerned that “an awful lot of misnomers are passed on from one paper to another, and actually you have to see the thing in the field… we had to learn the lessons ourselves.” Alongside personal observation, another respondent tried to make sense of the ash dieback outbreak by referencing previous outbreaks: “I was working around in the area, and I was looking at ash trees, and I thought, with the media coverage, I thought, actually this is really serious, this could be another Dutch elm disease” (R13). Another (R12) referenced an outbreak of Asian longhorn beetle (*Anoplophora glabripennis*) earlier that same year. This benchmarking of new risks against previous outbreaks, together with reference to the widespread loss of ash in Denmark and the media coverage, led to a consensus from our respondents that ash dieback could have a significant impact on British ash populations.

Echoing the reputational concerns reported above for OPM, some respondents felt that the nature and speed of the Government’s response to ash dieback could partly be explained by the need for officials to safeguard reputation by responding quickly to what was perceived to be acute public concern over ash dieback. Following Blok et al.’s (2008) contention that experts often attribute public anxiety to people’s emotional fears, several respondents in our study argued that public concern around ash dieback can be explained in subjective and emotional terms, with ash considered highly valued by UK publics as an important part of Britain’s natural cultural heritage. An outbreak manager (R6), for instance, suggested that dieback hit the headlines in the way it did “because it was Dutch elm disease all over again … I think it was really the emotional tug of losing another much loved aspect of the countryside and landscape.” Another respondent (R7) suggested that, alongside Dutch elm disease, the public perceived a failure by government to deal with other biosecurity risks, such as foot and mouth disease. A local authority respondent used migration as a heuristic device to further attribute public concern: “I think it really linked into this whole issue around migration and immigration and so on. I think it linked to a feeling that this was something that was imported, and is then killing our British tree stock.”

In summary, as with OPM, respondents utilised a range of information sources to construct their understanding of the risks posed by ash dieback. Beyond official risk assessments, respondents drew on what they perceived as public concern and the Government’s response to that public attention in the wake of a period of intense media attention when the outbreak was first identified. There was referral to past risk events and evidence of sensitivity towards maintaining reputation as scientists and policymakers attempted to deal both with, in SARF terms, the ‘risk event’ itself, alongside ‘the social construction of the risk’.

## 5 Conclusions

The two tree health case studies presented in this paper illustrate the extent to which expert risk perceptions are influenced by a range of socio-political, affective and cultural filters. We have identified the situated and socially-constructed nature of those risk perceptions through the roles played by such actors and have identified the ways in which risk judgements are constructed over time through a process of drawing on diverse information sources, social and cultural backgrounds and heuristic devices. In many instances, respondents indicated high levels of concern in the early stages of outbreaks when there was limited scientific evidence, a lack of clarity on management responsibilities or regulatory mechanisms.

The issue of uncertainty poses one of the greatest challenges facing experts in framing objective risk assessments for both current and future tree health outbreaks. For many tree pests and diseases, there is uncertainty about the likelihood of introduction and spread but also about the effectiveness of any attempts to control, manage or contain an outbreak once it is underway (Potter and Urquhart, 2017, Brasier, 2008). Furthermore, scientific understanding is often assembled incrementally as outbreaks unfold, making effective management and control very difficult to plan, justify and implement. Our case study outbreaks present different risk profiles, with OPM displaying a range of expert representations of the risk, many of which are shaped by institutional affiliation and the need to deal with the pest in order to safeguard public health. The nature and tenor of the response to ash dieback emerges as similarly complex, with risk managers having to assemble the risk case to justify action in the face of an incomplete and contested scientific evidence base. For policy makers and risk managers much of the early risk management in relation to ash dieback focused on dealing with the reputational risks at stake given an intense public and media scrutiny during the initial stages of the outbreak in 2012.

As with other risk controversies, those managing tree pests and diseases must manage not only risks to tree health (and in the case of OPM, human and animal health), but also be sensitive to the way publics might assess risk managers’ subsequent response (Ramsey, 2008, Leiss, 2003). Under conditions of uncertainty, policymakers and decision-makers may feel particularly exposed to risks to their reputation, partly due to the need to make and justify decisions early in outbreaks that may impose significant costs on a range of stakeholders and publics. Both scientists and outbreak managers dealing with pests and diseases described how they piece together information and evidence over time to build up a picture of the risk, through annual surveying and gathering data and observing the epidemiology and population dynamics of the organism (see Table A1). However, in addition to technical assessments of risk, such as PRAs, they need to assemble risk judgements from many different sources and in doing so may also attribute risk perceptions to other stakeholders and wider publics. Decision makers, therefore, may look to the media as a proxy for public concern but, as a number of our respondents recognised, while the media may help to inform the public and raise (a degree of) *awareness*, this does not necessarily equate to public *concern*.

Our analysis suggests that where there are concerns over uncertainty and reputational risk, decision makers are likely to be sensitive to what they believe the public is thinking. In the absence of reliable empirical evidence about public risk perceptions, they may therefore attribute risk perceptions to wider publics and other stakeholders. This observation suggests that the SARF needs to more fully recognise both the socially constructed nature of expert risk perceptions and to reconsider casting the role of the public as ‘amplifiers’ of risk. How experts attribute public concern about hazard events can either intensify or attenuate the perceptions of those charged with managing outbreaks or those blamed for new pest or disease incursions.

By opening the black box of how experts judge risk, our analysis suggests a need for recognition within SARF of the socially constructed nature of expert risk judgment and a reconsideration of expert assessments as the benchmark around which public perceptions are amplified. In the tree health case, we have shown expert risk perceptions to be heterogeneous and dynamic, drawing on a wide range of ‘evidence’, especially when there are high levels of scientific uncertainty. Institutions managing and regulating for outbreaks may, therefore, respond both to the hazard event itself but also to what they attribute as public concern in their efforts to ensure the social acceptability of any interventions.
