# Supplementary material for: Expert risk perceptions and the social amplification of risk: A case study in invasive tree pests and diseases
Source: Environ Sci Policy. 2017 Nov;77:172–8. doi: 10.1016/j.envsci.2017.08.020 (PMC5637881; doi:10.1016/j.envsci.2017.08.020)
Supplement: Supplementary file 1 [file mmc1.docx]

**Supplementary material: Appendix 1**

**Table A1: Respondent information sources of risk information**

| Scientists | Direct observation and scientific research;  Interactions across scientific networks nationally, across Europe and globally - including field visits, collaborative research and sharing knowledge in scientific papers and conferences;  Engagement with policymakers and risk managers through science advisory panels and commissioned research;  Awareness of media coverage of pest and disease outbreaks. |
| --- | --- |
| Policy makers | Official institutional channels. |
| Outbreak managers | Government policy and management strategies;  Personal observation in their day-to-day work, e.g. through direct management activities to monitor or control spread;  Anecdotal evidence from interactions with landowners and nursery owners. |
| Local authority officers | Personal experience and local observations of outbreaks;  Interactions with colleagues;  Wide range of local stakeholders;  Information from central government departments;  First alerts often via the news media or a report from a member of the public. |
| Public park or open space managers | Personal observation and experience;  Interactions with the FC, the Food & Environment Research Agency (Fera) and arboriculturists. |
| Foresters | Personal observation and experience;  Interactions with other foresters;  Networks across a range of non-governmental organisations, such as the Confederation of Forest Industries (Confor), the Royal Forestry Society (RFS), the Country Land and Business Association (CLA), Woodland Trust, National Trust, FC and Fera. |
| Plant or tree nurseries | Personal observation;  Interactions with colleagues & other nurseries; government officers (e.g. tree officers and plant health inspectors), trade associations;  Wider trade networks across Europe, such as via the European Nursery Federation. |
| Non-governmental organisations | Networks across private and public sector, including their own members. |
